# Evaluation of retinal nerve fiber layer thickness measurements using optical coherence tomography in patients with tobacco-alcohol-induced toxic optic neuropathy

**DOI:** 10.4103/0301-4738.60087

**Published:** 2010

**Authors:** Frederico C Moura, Mário L Monteiro

**Affiliations:** Neuro-Ophthalmology Service, University of São Paulo Medical School, São Paulo, Brazil

**Keywords:** Optical coherence tomography, papillomacular bundle, retinal nerve fiber layer thickness, tobacco-alcohol amblyopia, tobacco-alcohol optic neuropathy

## Abstract

Three patients with progressive visual loss, chronic alcoholism and tabagism were submitted to a complete neuro-ophthalmic examination and to retinal nerve fiber layer (RNFL) measurements using optical coherence tomography (OCT) scanning. Two patients showed marked RNFL loss in the temporal sector of the optic disc. However, a third patient presented RNFL measurements within or above normal limits, based on the Stratus-OCT normative database. Such findings may be due to possible RNFL edema similar to the one that may occur in the acute phase of toxic optic neuropathies. Stratus-OCT was able to detect RNFL loss in the papillomacular bundle of patients with tobacco-alcohol-induced toxic optic neuropathy. However, interpretation must be careful when OCT does not show abnormality in order to prevent diagnostic confusion, since overestimation of RNFL thickness measurements is possible in such cases.

Tobacco-alcohol-induced toxic optic neuropathy is a condition that occurs in heavy alcohol drinkers and tobacco smokers and causes progressive painless visual acuity loss and central or cecocentral scotoma. The optic disc initially appears normal, but marked temporal disc pallor and retinal nerve fiber layer (RNFL) loss in the papillomacular bundle occurs rather late in the course of the disease.[1] RNFL loss has been documented by scanning laser polarimetry in chronic tobacco and alcohol users.[2] However, scanning laser polarimetry showed low power to discriminate abnormalities in temporal and nasal sectors of the optic disc in a previous study.[3]

Optical coherence tomography (OCT) is a non-invasive technique for the acquisition of cross-sectional images of retinal structures from which estimates of the RNFL can be made.[4] The ability of OCT to provide quantitative and reproducible measurements of the thickness of RNFL has been demonstrated in experimental and clinical studies, both in glaucoma as well as in neuro-ophthalmic diseases.[5,6] Recently, Kee and Hwang[7] reported the first patient with tobacco-alcohol-induced toxic optic neuropathy that was evaluated with OCT. Although the patient had bilateral visual loss, the equipment did not indicate reduction of the RNFL.

The purpose of this paper is therefore to report three further cases of tobacco-alcohol-induced toxic optic neuropathy submitted to Stratus-OCT (Carl Zeiss, Meditec, Dublin, CA, USA) that allowed us to better understand the spectrum of RNFL measurement change as evaluated by OCT in patients with such optic neuropathy.

## Case History

### Case 1

A 51-year-old man presented with a gradual decrease in vision over two years. He was a heavy smoker (two packs a day) of cigarettes and had positive history for daily ethanol intake (1.2 L of beer/day). He had no history of other ocular diseases and was not using any medication. On examination, his best-corrected visual acuity was 0.3 in each eye. Slit-lamp examination, extraocular motility and pupillary responses revealed no abnormal findings. Intraocular pressure was 14 mmHg in the right eye and 16 mmHg in the left eye.

**Figure 1 - Case 1:**
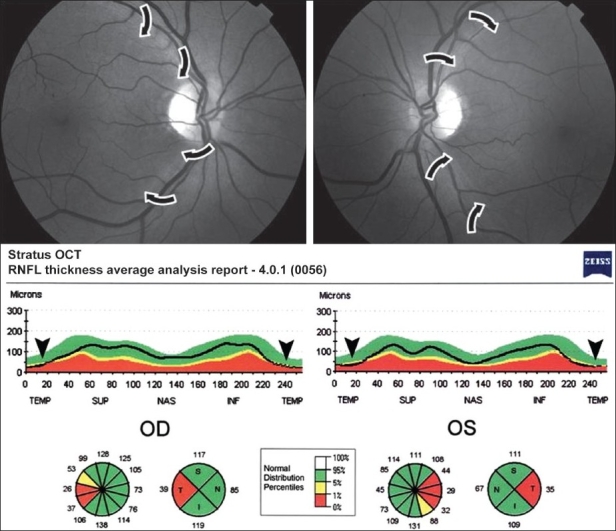
Above, Fundus photography showing temporal optic disc pallor and retinal nerve fiber layer loss in the papillomacular bundle of both eyes (arrows). Below, Stratus-OCT printout showing decrease in the retinal nerve fiber layer thickness involving the temporal quadrant of both eyes (arrowhead) and indicated as outside normal limits in the normative database display

Dilated fundoscopic examination disclosed severe temporal optic disc pallor and RNFL loss in the papillomacular bundle of both eyes [Fig. 1] and Goldmann visual field testing showed bilateral centrocecal scotoma. Stratus-OCT examination disclosed RNFL loss in the temporal quadrant in both eyes [Fig. 1].

### Case 2

**Figure 2 - Case 2:**
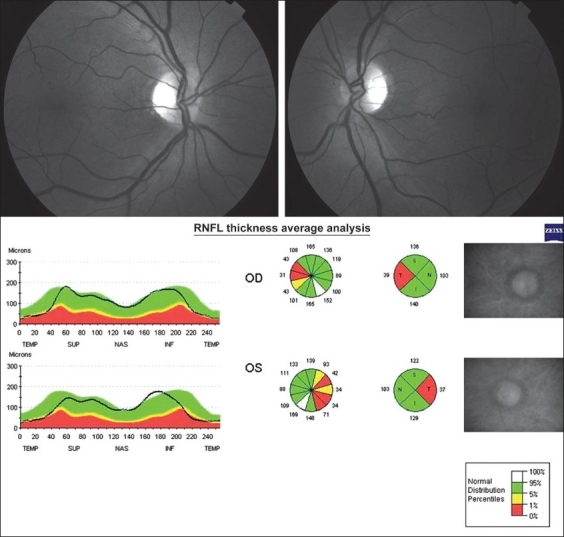
Stratus-optical coherence tomography showing retinal nerve fiber layer loss of the temporal quadrant in the normative diagram of both eyes (arrowhead)

A 52-year-old man presented with progressive decrease in vision of both eyes over one year. He had been smoking one pack of cigarettes a day for 15 years and used to take four to nine drinks of sugarcane-derived distilled beverage (41% alcohol content) per week. On examination, his best-corrected visual acuity was 0.8 in both eyes. His pupils were isocoric and sluggish to light stimulation. Intraocular pressure was 17 mmHg in the both eyes. Dilated fundoscopic examination showed bilateral temporal optic disc pallor and RNFL loss in the papillomacular bundle. Goldmann visual field testing showed bilateral centrocecal scotoma. Stratus-OCT examination disclosed RNFL loss in the temporal quadrant of both eyes and a small increase in the RNFL thickness at the 5 o'clock-hour meridian 30^−0^ segment in both eyes [Fig. 2].

### Case 3

A 39-year-old man presented with blurred vision in both eyes over a period of seven months. He had smoked one pack of cigarettes per day for the last 15 years and used to drink 200 ml of sugarcane-derived distilled beverage drink (41% alcohol content) per day over the last year. His best-corrected visual acuity was 0.2 in each eye. Pupillary examination was within normal limits.

**Figure 3 - Case 3:**
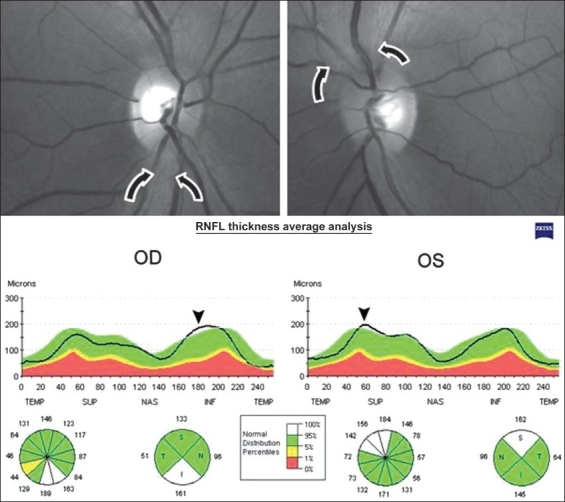
Above, Fundus photography. Note retinal nerve fiber layer swelling (arrows) and temporal optic disc pallor and retinal nerve fiber layer loss in the papillomacular bundle of both eyes. Below, Stratus-OCT printout showing increased retinal nerve fiber layer thickness in the inferior quadrant of the right eye and in the superior quadrant of left eye (arrowhead)

Dilated fundoscopic examination showed mild temporal optic disc pallor and RNFL loss in the papillomacular bundle of both eyes [Fig. 3]. Goldmann visual field testing showed bilateral central scotoma. Stratus-OCT examination showed a small decrease in the RNFL thickness of the inferotemporal quadrant and a markedly increased RNFL thickness in the inferior quadrant of the right eye. OCT disclosed only increased RNFL measurements in the superior quadrant of the left eye [Fig. 3].

## Discussion

Tobacco-alcohol-induced toxic optic neuropathy is a syndrome characterized by papillomacular bundle damage, central or cecocentral escotoma and progressive visual function loss. Although it has been classically considered to be optic neuropathy, the primary lesion site has not actually been localized to the optic nerve and may possibly originate in the retina, chiasm, or even the optic tracts.[8] The pathogenesis of tobacco-alcohol-induced toxic optic neuropathy is probably multifactorial but malnutrition associated with alcohol intake and heavy smoking are probably the most important factors.[1]

Two of our three cases with tobacco-alcohol-induced toxic optic neuropathy had a history of heavy alcohol intake and dietary deficiencies (Cases 1 and 2). In these cases, Stratus-OCT RNFL thickness of the temporal quadrant was decreased. This pattern is in agreement with RNFL loss documented by red-free retinography and with the visual defect seen in our patients. Our third case also had a history of heavy alcohol and tobacco use. In this case, however, despite the presence of temporal RNFL loss observed on ophthalmoscopy, Stratus-OCT did not reveal a correspondent reduction. On the contrary, it showed increased RNFL thickness measurements (represented as white color in the normative database display) suggesting RNFL edema, as reported by Kee and Hwang.[7] We agree with those authors that in the early stages of toxic optic neuropathies, disc and RNFL edema may be seen in some patients, especially in acute poisonings like methanol poisoning. Such occurrence may be the reason for overestimation of RNFL thickness measurements with resultant absence of temporal RNFL loss in the Stratus-OCT printout of our Case 3. Although visual loss was not acute when we examined him, optic neuropathy would probably be active due to uninterrupted alcohol ingestion.

In conclusion, Stratus-OCT assessment --is capable of identifying RNFL loss in the papillomacular bundle of patients with late-stage tobacco-alcohol-induced toxic optic neuropathy. However, in some cases, the technology may fail to show RNFL defect even in patients with visual loss of more than one year duration, presumably because the RNFL defect is masked by RNFL edema. Therefore, clinicians must be careful when interpreting the Stratus-OCT printout and be aware of the possibility of RNFL measurement overestimation in order to avoid diagnostic confusion in patients with tobacco-alcohol optic neuropathy.
